# Emerging Importance of Tyrosine Kinase Inhibitors against Cancer: Quo Vadis to Cure?

**DOI:** 10.3390/ijms222111659

**Published:** 2021-10-28

**Authors:** Raj Kumar Mongre, Chandra Bhushan Mishra, Arvind Kumar Shukla, Amresh Prakash, Samil Jung, Md Ashraf-Uz-Zaman, Myeong-Sok Lee

**Affiliations:** 1 Molecular Cancer Biology Laboratory, Cellular Heterogeneity Research Center, Department of Biosystem, Sookmyung Women’s University, Hyochangwon gil-52, Seoul 04310, Yongsan-gu, Korea; Rajkumar_Mongre@urmc.rochester.edu (R.K.M.); jj-31@hanmail.net (S.J.); 2 Department of Microbiology & Immunology, David H. Smith CVBI, University of Rochester Medical Center, 601 Elmwood Avenue, Rochester, NY 14642, USA; 3 Department of Pharmacology & Chemical Biology, Baylor College of Medicine, Baylor Plaza, Houston, TX 77030, USA; chandra.medicinalchemist@gmail.com (C.B.M.); ashrafbd87@gmail.com (M.A.-U.-Z.); 4 School of Biomedical Convergence Engineering, Pusan National University, Yangsan 50612, Gyeongsangnam-do, Korea; arvindkumarshuklapnu@gmail.com; 5 Amity Institute of Integrative Sciences and Health (AIISH), Amity University Haryana, Gurgaon 122413, India; aprakash@ggn.amity.edu

**Keywords:** receptor tyrosine kinases, drug resistance, targeted therapy, cancer, mutation, clinical trials

## Abstract

GLOBOCAN 2020 estimated more than 19.3 million new cases, and about 10 million patients were deceased from cancer in 2020. Clinical manifestations showed that several growth factor receptors consisting of transmembrane and cytoplasmic tyrosine kinase (TK) domains play a vital role in cancer progression. Receptor tyrosine kinases (RTKs) are crucial intermediaries of the several cellular pathways and carcinogenesis that directly affect the prognosis and survival of higher tumor grade patients. Tyrosine kinase inhibitors (TKIs) are efficacious drugs for targeted therapy of various cancers. Therefore, RTKs have become a promising therapeutic target to cure cancer. A recent report shows that TKIs are vital mediators of signal transduction and cancer cell proliferation, angiogenesis, and apoptosis. In this review, we discuss the structure and function of RTKs to explore their prime role in cancer therapy. Various TKIs have been developed to date that contribute a lot to treating several types of cancer. These TKI based anticancer drug molecules are also discussed in detail, incorporating their therapeutic efficacy, mechanism of action, and side effects. Additionally, this article focuses on TKIs which are running in the clinical trial and pre-clinical studies. Further, to gain insight into the pathophysiological mechanism of TKIs, we also reviewed the impact of RTK resistance on TKI clinical drugs along with their mechanistic acquired resistance in different cancer types.

## 1. Introduction

Over the past 250 years, we have seen numerous revolutionary innovations in the war against cancer. However, the causes of cancer are not completely understood to date. Cancer is a result from genetic mutations that lead to uncontrolled propagation of a normal cell [1]. It interferes with the normal cellular program, initiated by the primary tumorigenic site and disseminated to the metastatic site [2]. The current treatment options of cancer are chemotherapy, radiation therapy, combination therapy, laser therapy, and surgery, which often also destroy normal cells and trigger severe side effects in the person with a high burden of the tumor. Therefore, it would be desirable to develop novel therapeutic strategies that can selectively target cancerous cells.

Currently, approximately 90 different tyrosine kinases (TKs) or receptor tyrosine kinases (RTKs) have been identified and further categorized into 20 different subfamilies based on receptors and their ligands [3,4,5,6,7,8]. RTKs are enzymes that play critical roles in most fundamental cell programs in normal cells, including cell proliferation, cell cycle, migration, metabolism, programmed cell death, survival, and differentiation [9]. In contrast, these RTKs catalyze the transfer of gamma-phosphate of ATP to tyrosine hydroxyl groups on target proteins. This posttranslational modification is a crucial phenomenon for cellular communications and homeostasis in normal cells [3,4,5,6,9]. However, RTKs are abnormally implicated in diverse carcinogenic expansion and progression [3,4,5].

Further, RTK associated signaling cascades are able to mediate dysregulated propagation of cells and contribute to sensitivity towards apoptotic stimuli, especially resistance against drugs [6]. In cancer cells, these cascades are epigenetically altered to provide selective advantage. To date, there are more than fifteen different classes of RTKs that have been well explored, with their function and origin shown in Table 1 [9]. EGF receptor/ErbB family, which belongs to RTK class I, is a key mediator in a diverse cellular mechanism including regulation of homeostasis [3,10]. However, the deficit of these ErbBs led to the induction of neurodegenerative diseases and embryonic lethality [10]. On the other hand, elevated levels of ErbB/EGFR are associated with the high grade of tumors [10]. FGFR are also known as an immunoglobulin superfamily, involved in the interaction with various proteins and produced by macrophages [8]. These FGFRs have a unique role in the development of normal cells. Any abnormal levels of FGFs lead to several developmental defects and are linked with angiogenesis, keratinocyte and wound healing processes, and pluripotency via the Wnt signaling pathway [8]. VEGFs are involved in the formation of blood vessels, and permeability and gain or loss results in diabetes, ischemic stroke, and cancers [11]. Another RTK, RET, is also associated with hereditary cancer multiple endocrine neoplasia type 2 [12]. Due to gain or loss of function, deletions, insertions, amplification, point mutation, and elevation in RTKs resulted in dominating oncoprotein translation and malfunctioning of the signaling network that led to onconeogenesis [3,4,5,6].

The breakthrough innovation of RTKs in treating different cancers has become an attractive target, superior to other traditional therapies in selectivity, efficacy, and safety. Drug molecules that inhibit carcinogenesis by binding specifically to their respective targets are the foundation of targeted cancer therapy. To date, a lot of work has been conducted to find synthetic small chemical inhibitors of RTKs to inhibit them selectively [3]. These RTKs play a vital role in regulating cell growth and differentiation, proliferation, motility, adhesion, and apoptosis [4,5,6]. Many TKI drugs are being tested in clinical trials for the treatment of cancers. Clinical results showed that cancer treatment with effective TKIs tends to achieve efficacy, particularly with overcoming TK-resistance (Tables 1 and 2). On the other hand, the non-receptor tyrosine kinases relay intracellular signaling transduction, mediated by dimerization of ligands binding site which collectively induces cytoplasmic domains autophosphorylation of RTKs [5,6,7,8]. Numerous cytoplasmic signaling routes exist, including the Ras/Raf mitogen-activated protein kinase pathway, the Akt pathway/protein kinase -C pathway, the transcription-3 pathway, and the scaffolding proteins mediated signal cascades, which are well reported in several disease models [13,14,15,16].

Chemotherapy is considered a promising option for the treatment of cancers. However, 90% of chemotherapy failures were observed in the invasion and dissemination of malignancies due to drug resistance. A substantial percentage of patient tumor cells turned into resistant cells that create severe problems in patients. [17,18,19]. Apart from this, numerous complications have been shown in antitumor remedies, such as cytotoxic chemotherapy and targeted therapy resistance [20].

**Figure 1:**
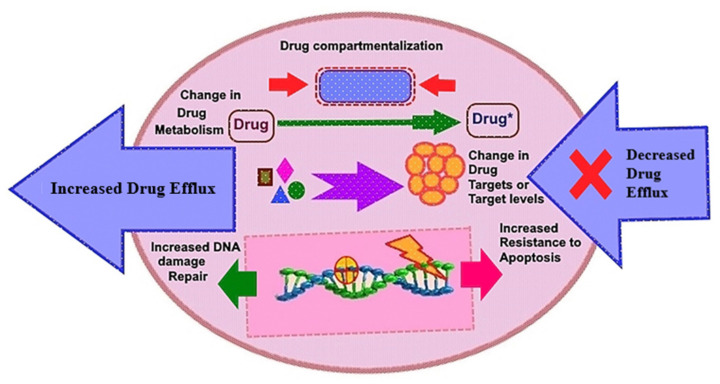
Depiction of the fundamental drug resistance pathways in cancer cells. Drug efflux mediated cellular programming as drug inactivation, multi-drug resistance, apoptosis suppression, changes in drug metabolism, epigenetic modifications, drug targets, accelerated DNA-repair, and target gene amplification by which cancer cells become resistant to various drugs.

To date, there are few promising approaches to control cancer progression based on molecular targets: (1) kinase inhibition at the translational level, (2) immunotherapy to enhance cancer’s rapid immunological defense mechanisms, (3) improvements in treatment choices, (4) delivery of a targeted medicine into proliferating tumor cells, and (5) minimization of the detrimental consequences of chemotherapeutic drugs, among other things [17,21,22]. Chemotherapy resistance is produced by a number of mechanisms, including multidrug resistance (MDR), drug efflux, drug inactivation, cell death prevention (apoptosis suppression), epigenetic, drug metabolism, DNA repair, and gene amplification (Figure 1). We discuss the many anticancer drugs based on tyrosine kinase inhibitors (TKIs) as well as the underlying mechanisms of acquired resistance to RTKI therapy. Non-RTKs, ErbB/HER family members, BCR-ABL, platelet-derived growth factor receptor-alpha/beta (PDGFR-α/β), and vascular endothelial growth factor receptors (VEGFRs) are all modulated by imatinib. Other RTKIs, such as trastuzumab, which targets the HER2/ErbB2 receptor, cetuximab, erlotinib, and gefitinib, which targets the EGFR, also effectively impede cancer growth.

## 2. Basic Concept on Targeting Receptor Tyrosine Kinases: Effective Strategies to Cure Cancer

The human kinome comprises more than 518 kinases with specific roles based on their subfamilies. Approximately 90 tyrosine kinases and more than 40 tyrosine kinase-like genes control cellular programming and homeostasis [23]. Briefly, tyrosine kinases (TKs) are enzymes involved in transferring phosphate from the high-energy giver ATP to tyrosine residues (serine/threonine) into target amino acids. The use of ATP results in the triggering of intracellular signaling pathways, which closely govern survival, differentiation, and cell development. Certain tyrosine kinase receptors are essential for normal cell regulation and function. However, its dysregulation promotes the growth of tumors and blood vessels via tumor angiogenesis [24,25]. These kinases are generally present as monomers on the cell surface which bind with different growth factors to initiate kinase activities, auto-phosphorylation, and dimerization [26,27]. RTKs such as Met, Axl, Kit, and EGFR are altered in most cancers [28,29]. The proteins PDGFR, VEGFR, and fibroblast growth factor receptor (FGFR) are related to promote metastasis and angiogenesis under tumor microenvironment [30]. Additionally, VEGFRs are expressed on vascular endothelium, and the interactions of VEGF–VEGFR are crucial for the development and propagation of endothelial cells [31,32,33]. It is also well reported that the synergistic effect of fibroblast growth factor with VEGF accelerates vasculature mediated angiogenesis in cancers [34].

**Figure 2:**
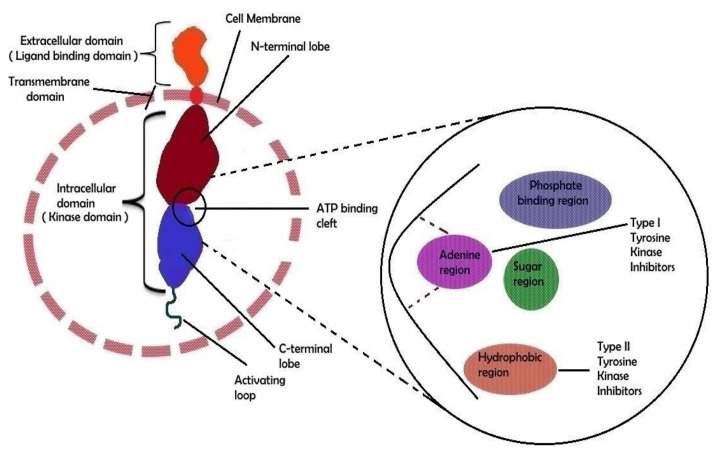
Molecular structural feature of RTK. A receptor tyrosine kinase’s extracellular domain can bind particular ligands such as growth factors, whereas the intracellular domain is responsible for the kinase’s (auto)phosphorylation. The external and internal domains are separated by the transmembrane region which is fixed in the cell membrane. The ATP-binding cleft is located between the two lobes of the intracellular domain. A schematic depiction of the ATP binding cleft with its numerous regions is shown on the right side of the image. Type I and type II tyrosine kinase inhibitor binding sites have been shown in biochemical general structure model.

RTKs are required for extracellular signal cascades entering the cell, whereas non-RTKs are responsible for internal communication. [34]. The RTK monomer is made up of N-terminus an extracellular ligand-binding domain, C-terminus, and a transmembrane domain containing action of tyrosine kinase (Figure 2). The kinase domain is bi-lobar, with an ATP binding cleft between the N- and C-terminal lobes [9,35]. The ATP-binding site is divided into three sub-regions: sugar binding, adenine binding, and phosphate binding [36]. Kinases’ C-terminal lobes are made up of an activation loop that is defined by a certain amino acid combination at the start of the loop. This combination is known as the ‘DFG motif, composed of the amino acids aspartic acid, phenylalanine, and glycine, denoted as D, F, and G, respectively [35]. 

The hydrophobic pocket of RTKs is recognized by the subclass of RTK inhibitors that will be addressed in the latter section. Ligand binding to the receptor’s extracellular domain promotes receptor dimerization, which leads to auto-phosphorylation of certain tyrosine residues in the cytoplasmic kinase domain [37]. Additional kinase phosphorylation sites are used to govern a protein–protein interaction for regulating their own kinase activity. When the receptor is activated, it assigns a specific phosphorylation site to the linked proteins [37]. Phosphorylation of respected protein activates numerous signaling pathways, which eventually play a crucial role in biological responses [9]. 

## 3. Mutations in RTKs Associated with Diverse Mutational Pathways

**Figure 3:**
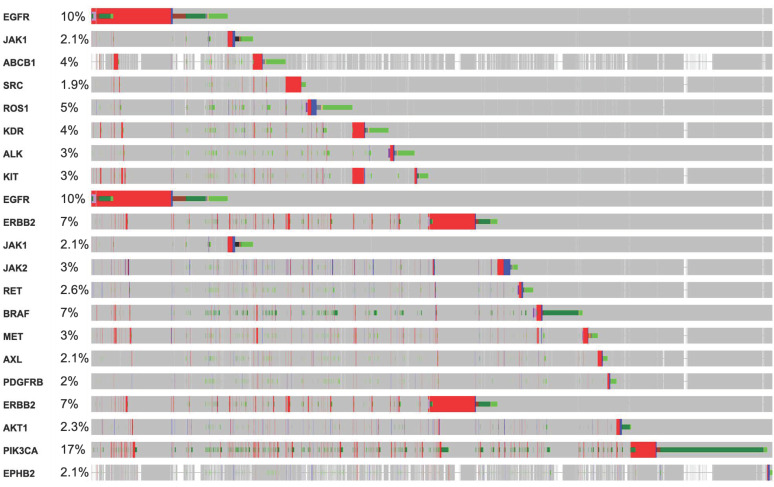
OncoPrint analysis showing a frequency of mutations in RTKs, and other oncodrivers which suggest a multi-genomic alteration event in various cancers. Among the main RTKs, KRAS, PTEN, BRAF, EGFR, and ERBBs were the most frequently mutated in malignancies.

RTKs are master key regulators in several diseases, including cancers [4,5,9]. Considerable research has already been undertaken over the last decades to understand the cellular mechanism associated with RTKs. However, mutations in RTKs have attracted significant attention in cancer research [37,38,39]. Figure 3 shows different % mutations of RTKs in several cancers and other oncodriver′s proteins. Here, we summarize a few major pathways that are linked with mutation-induced cancers.

### 3.1. Alteration in RTKs Modulate Carcinogenesis via RTK-RAS Mutational Pathway

**Figure 4:**
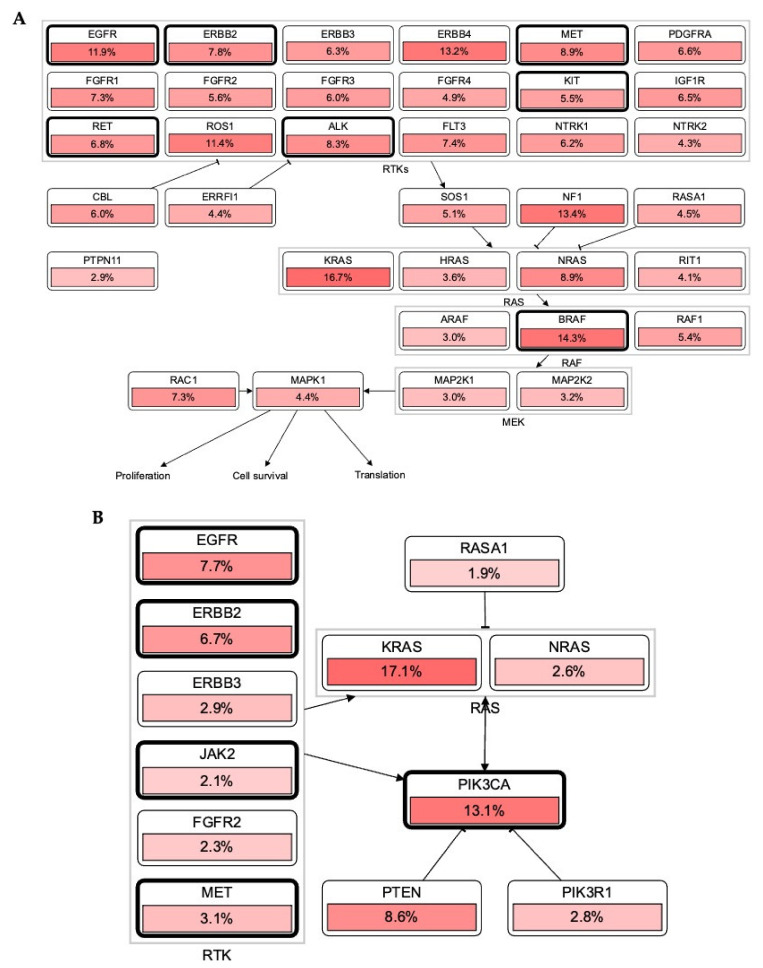
RTKs involvement in the RAS carcinogenic pathway. (**A**,**B**): Most RTKs in different malignancies have undergone mutational alterations (data was retrieved from cBioPortal patient cancer databank). In each pathway, RTKs including other oncodrivers were highly mutated and modulated a number of oncogenic mechanisms which promote tumor initiation, invasiveness, and tumor burden spread in several cancers.

RTKs and their downstream affecters, such as RAS, appeared as important machinery in the pathogenesis of several cancers [14]. Germline and acquired mutations have been involved in most of the cancer initiation. Constitutive activation of intrinsic receptors by mutation in EGFRs, ERBBs, PDGFRs, and other RTKs has a pivotal role in controlling tumor burden and patients’ survival [15]. Chou et al. demonstrated that 61.1% of mutations were found in EGFR, where the disease control rate and the response rate were 68.5 and 56.8%, respectively [40]. In line with this, the cBioPortal database OncoPrint analysis shows that most of these RTKs have the highest alteration rate, such as EGFR = 10%, ERBB2 = 7%, BRAF = 7%, ROS1 = 5%, and MET = 3%, in 165 (44,752 patients/46,632 samples) cancer cohort studies (Figure 3). Collectively, these mutational changes have exhibited a significant reduction in disease-free and overall survival of patients with cancers. However, negative for EGFR mutations showed better overall survival (OS, *p* = 0.046) and progression-free survival (PFS, *p* = 0.011). Additionally, the cBioPortal database RTKs-KRAS-BRAF mutational pathway also revealed an aberration in RTKs which simultaneously induces 16.7% mutation in KRAS, associated with 14.3 and 13.4% mutational changes in BRAF and NF1, respectively. Together these mutational changes alter cancer cell propagation, translation, and survival via the RAS-BRAF mediated signaling pathway (Figure 4A).

### 3.2. Association of RTKs via KRAS-PIK3CA Pathway in Cancer

The most altered genes were KRAS with 17.1% in 44,752 patients (46,632 samples in 165 studies) in the KRAS-PIK3CA associated pathway. RAS has been known over the last three decades, and its mutation acts as a driver for cancer progression and maintenance [41]. Jang et al. reported a multigene panel study of mutations where KRAS, NRAS, BRAF, PIK3CA, TP53, and POLE were most mutated in 90 colorectal carcinoma patients [42]. Further, EGFR inhibitors such as erlotinib and gefitinib are beneficial in the management of lung cancer. However, it also revealed limited efficacy in different patients. Patients with KRAS mutations have also been associated with resistance to the EGFR inhibitors. As a result, there is an urgent need to investigate new hybrid small molecules to alleviate tumor burden in patients with KRAS-EGFR mutations. In accordance with this, KYA1797K demonstrated significant effectiveness against KRAS-driven cancer in KrasLA2 mice via inhibiting the Ras-ERK pathway. The destabilization of Ras via RTKs can be a promising strategy to halt a tumor in *KRAS*-mutated NSCLC [43]. 

**Figure 5:**
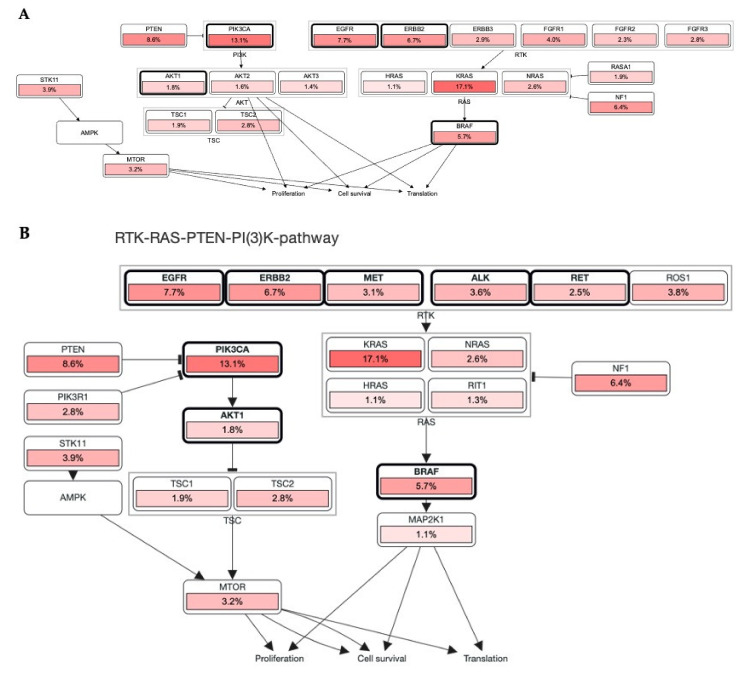

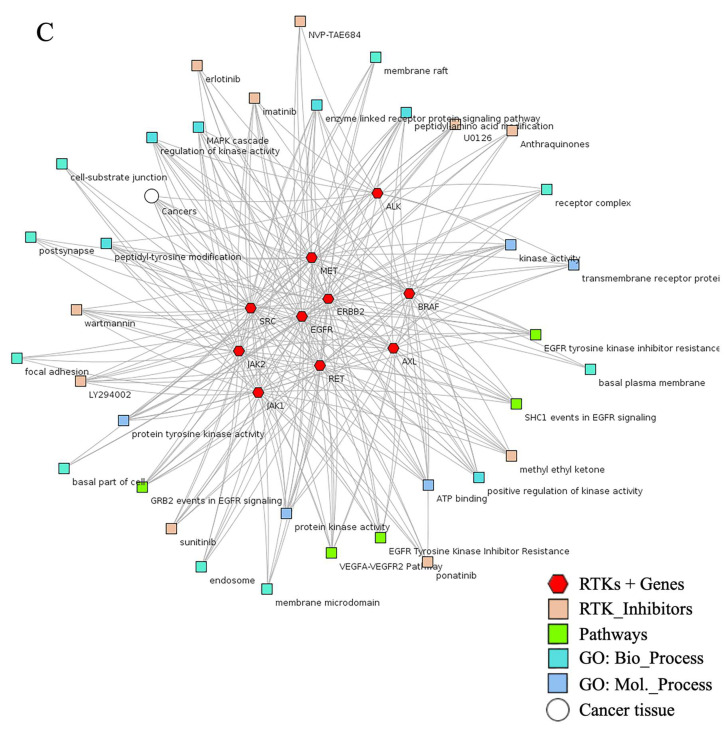
Mutation of RTKs caused mutation in KRAS, PIK3CA, BRAF, PTEN, and mTOR ablation that induced tumorigenesis. (**A**,**B**) Mutational changes occurred in most RTKs, including EGFR, ERBB3, MET, ALK, RET, and FGFRs, which involved diverse mediators that induced cell proliferation. Pathways retrieved from cBioPortal patient cancer databank. (**C**) Association of RTKs with their inhibitor and major GO were visualized by an abstracted network analysis.

Lewis Cantley discovered the PI3Ks as an unknown phosphoinositide kinase correlated with the polyoma middle T protein [44]. It participates in most of the cellular signaling, including cancers. An elevation or mutation in PIK3CA led to EGFR upregulation and ERK mediated paracrine cascades in breast cancer [45]. In line with this finding, it has been proposed that EGFR inhibitor treatment regulates overall and disease-free survival of NSCLC patients with the EGFR mutation coexisting with the PIK3CA double mutation [46]. In Figure 2, PIK3CA with 13.1% mutation intricately involved in the progression and pathogenesis of cancers via AKT1 (1.8%), mTOR (3.2%), and BRAF (5.7%) mediated proliferation and survival of cancer cells. It also showed that these RTKs directly induced PIK3CA upregulation and mutation, which augmented tumorigenesis (Figure 4B). The significant role of RTKs has been shown in the RTKs-PTEN-KRAS-MTOR and RTKs-PTEN-PIK3CA-AKT1-KRAS mutational pathways and augment translation, proliferation, and survival of tumor cells (Figure 5A,B). Additionally, binding of these RTKs with inhibitors control several cancers via diverse mechanisms (Figure 5C). Collectively, RTKs could be involved in resistance to the drug in cancer cells, and their inhibition may be exploited in target therapies.

## 4. Binding Site Architecture of Tyrosine Kinase

In eukaryotic, approximately 2% kinase domains catalyze the phosphate (PO_4_^3−^) transfer of ATP to a particular substrate of hydroxy-tyrosine by posttranslational modification [47]. Due to the pivotal role of tyrosine phosphorylation via kinase domains, it makes them highly conserved in diverse diseases [3,4,5,6,9,35,48,49]. In EGFR (PDB: 1M14), these domains are made up of a C-lobe (larger) and N-lobe (small). Among them, there are five, the αE, αF, αG, αH, and αI helix, which are found in the large C-lobe. The β1 to β5 five beta sheets are positioned in the N-lobe with one α-helix (αC, amino acid residue 729-744). ATP binding sites are mostly in a deep cleft of both C- and N-lobes and in a highly conserved glycine-rich phosphate-binding loop that bridges β1 with β2 in the N-lobe [50]. The phosphates of ATP significantly interact with a high glycine-rich loop of EGFR [51]. In addition, conserved glutamate (Glu738) in EGFR of the αC helix is formed by actively ion association with a Lysine (Lys721) in β3 of N-lobe, adding α and β phosphates of ATP (Figure 4). In the C-lobe, it creates the ATP-binding cleft with a highly conserved Asp812-Asn818 catalytic loop, among which the interaction of Asn818 by hydrogen bond places Asp812; however, Asp812 binds with the hydroxyl chain of tyrosine substrate [47,51]. Asp831-Val852, together, form an activation loop in the C-lobe that consists of base Asp-Gly-Phe and Asp831-Gly833 DFG motif [51]. The phosphorylation of C-terminal domain containing Tyr974, Tyr992, Tyr1048, Tyr1068, Tyr1086, Tyr1101, and Tyr1173 shows auto-catalytic activities [47]. In contrast, phosphotyrosines residue of the C-terminal is considered for the EGFR-drug or small inhibitors docking site, Src homology, and PTB binding domain. The crystal structure of the EGFR kinase domain, which contains forty amino acids that take a substantial role in the development of EGFRK-specific inhibitors such as erlotinib (OSI-774, CP-358,774, TarcevaTM), is currently in Phase III clinical trials as an anticancer treatment [52]. The phosphorylation of EGFR is linked to a number of downstream pathways, including the RAS-RAF-MEK-ERK and the PI3K-AKT-mTOR circuit (Figure 2 and Figure 3).

**Figure 6:**
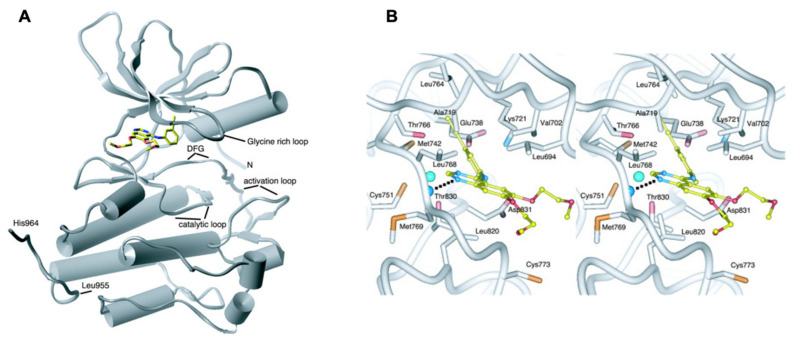
EGFR-erlotinib binding structure: Erlotinib binds at the gap between the EGFR kinase domain’s amino- and carboxy-terminal lobes (PDB: 1M14). (**A**,**B**) A stereo image of the inhibitor binding site and surrounding EGFRK/erlotinib residues. An H-bond from the Met769 amide nitrogen to erlotinib is indicated by a dashed line (It was procured from Stamos et al. [52]).

**Figure 7:**
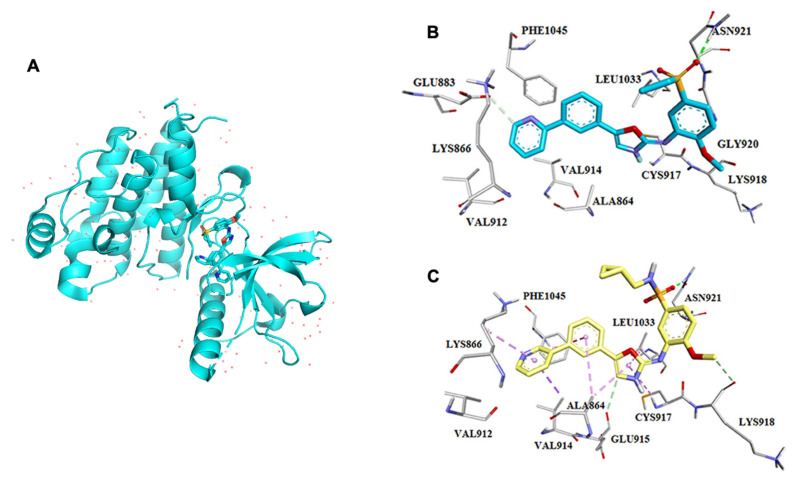
VEGF structure and interaction with its inhibitor pazopanib. (**A**) Structure of VEGF, (**B**) PDB: 1Y6A, (**C**) PDB: 1Y6B. These docked images were taken from Harris et al., 2005 [54] and 2008 [55].

Muller and his team reported the crystal structure of VEGF, and they found that it contains a cystine knot connected with two disulfide bonds between C51 and C60. It comprises four β (1,3,5,6) sheets linked by a cystine bond at the terminal end [53]. In β-sheet, β3 and β5 are connected by a hydrogen bond. Another segment of VEGF has α-helix and short β-strand in loop-1; here, loop-1 augments the binding between β1 and β3. Short β-strand, β5, and the beginning of β6 collectively create a three-stranded β-sheet near the cysteine knot. A hydrophobic core was created by combining a three-stranded-sheet, loops L1 and L3 (5–6), and helix 2 with the opposite monomer’s amino terminal -helix. This core is involved in bringing the four-stranded central sheet into equilibrium [53]. Harris et al. released an article detailing the discovery of pazopanib in 2008 [54]. That was a wonderful example of how homology modeling and SBDD were utilized to identify a previously available medication. Because the crystal structure of VEGFR2 was not known at the time, a homology model of the enzyme based on FGFR crystal structures was constructed to predict the binding mechanism of dimethoxyquinazoline analogs. The pyrimidine and quinazoline bound similarly at the ATP binding site, establishing hydrogen bonds with the backbone’s Cys919 residue (Figure 6). The crystallization of these compounds with VEGFR2 was confirmed in silico (PDB: 1Y6A, 1Y6B). In contrast, structural architechture of VEGF also depicting binding sites and its interaction with inhibitor pazopanib in several cancer cells (Figure 7). Finally, pazopanib was discovered when a series of novel analogs were developed, synthesized, and evaluated in vitro [54,55,56].

## 5. Major Classes in RTKs: Master Regulators of Cell Fate and Homeostasis

Since finding the first RTK, it has been divided into more than twenty subfamilies. The EGF receptor family and ErbB family are under RTK class I [57]. In RTK class II, insulin (IGF) associated receptor families play in diverse pathophysiological cascades that regulate propagation and trans-differentiation of a single cell [57,58]. Dysregulation of IGF-related RTKs has been linked in multiple diseases and tumor initiations [59]. The PDGF receptor family of the third class has a unique contribution in developing connective tissue cells and synthesis of organs [60]. The VEGFR group (class IV) is involved in the growth of endothelial cells and permeability of blood vessels. Dysfunction of VEGFR leads to oncogenesis by angiogenesis and dissemination of secondary metastatic tumors [61]. Apart from that, most of the other RTKs also participate in several cellular mechanism-mediated diseases. To date, numerous small molecule inhibitors were discovered targeting these RTKs with great efficacy in ailments, infection, and malignancies (Table 1).

## 6. Selective TKs Inhibitors in the Clinical Trial

RTKs emerged as a key player in most of the cellular processes. Alteration in RTKs resulted in the initiation of cancer growth and other diseases [9,73]. The EGFR, known as HER1, is from the ErbB group and is involved in diverse mechanisms, including invasion and dissemination of tumor cells [15,20,39,43,45,46]. Inhibition and targeting EGFR have undergone extensive studies to date, with the development of four-generation inhibitors, and it has been proved indispensable in the curing of EGFR-mutant patients [74]. First generation inhibitors, such as geftinib, erlotinib and icotinib, have shown significant antineoplastic activities in EGFR-mutant NSCLC patients [75]. Second-generation EGFR inhibitors, such as afatinib and dacomitinib, have been discovered with high affinity binding to EGFR, which are considered pan-HER inhibitors that significantly enhance survival [76]. However, these inhibitors also showed toxicity towards normal adjacent cells, limiting their progress in clinical trials. So, exploration of novel TK inhibitors with minimal toxicity is needed. However, several effective TKs inhibitors have been developed to date, and some of them showed promising anticancer activity in clinical trial studies (Table 2 and Table 3).

## 7. Recent Updates on Selective Tyrosine Kinase Inhibitors in Pre-Clinical Studies

RTKIs are efficient treatment options for RTKs sensiting-mutant cancers [82]. Abnormal changes in RTKs are responsible for tumor growth and invasion. Elevated levels or mutation of these TKs cause drug resistance and enhance tumor burden and invasion [82,83]. Although many small molecule inhibitors are already developed and in use now, these medicines have significant adverse effects, such as skin rash and gastrointestinal toxicity, thus limiting their therapeutic use. Numerous molecules have been designed and synthesized to discover more effective and safe inhibitors, showing promising anticancer activity in pre-clinical studies.

Li and his team reported pyrimidine derivatives containing cyclopropyl moiety, which exhibited admirable kinase inhibitory activities against EGFR double mutation (L858R/T790M) with an IC_50_ = 0.26 nM [84]. Similarly, Quinazoline based small molecule comprising benzazepine moiety also reduced the tumor growth with IC_50_ ranging from 1.06–3.55 μM by targeting EGFR-T790M [85]. Additionally, Pyrazole moiety has been inserted in many FDA-approved drugs such as Pazopanib, which offered more potent inhibitors. These pyrazole derivatives exhibited potent tumoricidal action against EGFR-tyrosine kinase with an IC_50_ of 395.1, 286.9, and 229.4 nM, respectively, compared to the standard drug, erlotinib [86]. The pyrazoline derivatives consisting of carbothioamide group showed significant EGFR inhibition, showing IC_50_ values ranging from 1.66 to 1.9 μM, and halted the growth of cancer cells [87]. Our previous studies also suggested that molecules from pyrazolo[3,4-*d*]pyrimidine, urea, piperazine, and hybrids have shown fabulous anticancer activities via inhibition of various oncoproteins against cancers [87,88,89]. These studies may help to postulate new possibilities for developing novel small molecule drugs with effective anticancer activity.

According to new research, VEGF is a critical activator of both normal and pathological angiogenesis [90]. Inhibition of VEGF mediated signaling by small molecules or antibody has been studied to halt cancer progression. Hennequin et al. identified 6,7-dimethoxyanilinoquinazolines (ZD4190) as a promising VEGF selective inhibitor with KDR/FTK potency ratio ranging from approximately 30-8000-fold [91]. Similarly, Quinazolin-4(3H)-one inhibited VEGFR-2 with an IC50 value of 0.340 ± 0.04 µM, which was higher than that of the reference medication, sorafenib [92]. Biphenylurea/thiourea derivatives tethered with heteroarylsulfonamide motifs were also found to be novel VEGFR2 inhibitors with significant inhibition compared to sorafenib [93]. Novel 2 indolinone-thiazole hybrid molecules effectively bound at the ATP binding site of VEGF-2 and showed a tumoricidal action with an IC_50_ value of 3.9 ± 0.13 µM, greater than Sunitinib [94]. Apart from that, many peptides-based pan-VEGF inhibitors with druggable binding sites might be crucial for discovering novel and selective VEGF inhibitors to manage the burden of cancer growth [95].

Among tyrosine kinases, the IGF system plays a pivotal role in the maintenance of cell growth and homeostasis [96]. It consists of TKs, insulin receptors (IRs), IGF1R, IGF2R, non-TKs, and some ligands IGFBPs, insulin, IGF1, and IGF2 [96]. However, pharmacological targeting kinases VEGFs may lead to more significant toxicity. Hence, the development of novel multitarget inhibitors that block IGF1Rs is immediately indispensable. One of the most potent IGF-1R inhibitors, phenylpyrazolo[3,4-*d*]pyrimidine, showed fabulous antitumor activities in the NSCLC induced xenograft and allograft model [97]. Gadekar and colleagues proposed 2,3-dihydroimidazo[2,1-*b*]thiazoles as dual EGFR and IGF1R inhibitors with reasonable drug likeness properties [98]. Tyrphostin AG 1024, a selective inhibitor of IGF-1R, markedly increases the response of tumor cells to ionizing radiation [99]. Moreover, Pyrazole pyridine-peperidinyl containing propanone also showed highly antitumor potency against cancer cells growth [88,100]. BMS-754807 is a reversible and strong inhibitor of the IGF-1 receptor/insulin receptor family kinases with Ki = 2 nmol/L [101]. It is currently in phase I clinical trial to treat a variety of human cancers [101]. Hence, we have seen the development of several IGFRs inhibitors; however, there is still an urgent need to design and synthesize more potent inhibitors that may be useful for managing cancers.

PDGF group kinases are essential for tumor growth and ablation. It is associated with a higher grade of tumor burden in ER + BRCA [30,65]. Therefore, blockade of PDGF would be one of the most promising strategies to mitigate the invasion of BRCA. In line with this, 3-substituted Quinoline derivatives were found to be effective PDGF inhibitors with IC_50_ value of 0.340 ± 0.04 µM [102]. Yang et al. found 2-pyrrolidone-fused (2-oxoindolin-3-ylidene) methylpyrrole derivatives that exhibited significant inhibitory activities against PDGFRβ in order to halt angiogenic tumor growth [103]. TK kinase FGFR elevation is also involved in the initiation of the cancerous tumor. Inhibition of FGFR by newly synthesized inhibitor JNJ-42756493 increases survival in patients with advanced or high-grade refractory solid tumors [104]. Similarly, PRN1371 exhibits prolonged target inhibition and highly selective covalent inhibitor against FGFR [105]. Apart from these inhibitors, several active synthesized small molecules efficiently block the translations of other TKs and halt oncogenesis.

Lastly, for the first time, a novel paradigm in which chemo and immunotherapy have been combined to treat cancer seems to be a wonderful antibody–drug conjugate (ADC) technique [106]. In contrast, a particular antibody can bind with an oncoprotein target without harming innocent adjacent cells, improving the efficacy of the drug. To put it another way, ADCs deliver a deactivated drug molecule to cancerous cells. The cytotoxin is produced after internalization of ADC to target cancer cells in which it plays a tumoricidal role. Currently, few ADC based potent drugs are available to cure high burden tumors, especially breast cancers, such as Roche’s Kadcyla (antiHER2 IgG1, trastuzumab, microtubule inhibitory medicine DM1), Gilead Sciences Trodelvy (IgGk–Sacituzumab govitecan-hziy), and Daiichi’s Enhertu (mAb-trastuzumab deruxtecan) [107]. Collectively, RTKs associated ADCs might be efficient and promising drug candidates which can target cancer cells without harming the healthy cells. It has been considered that it is capable to overcome the major clinical barrier of conventional chemotherapy, expanding a novel therapeutic window.

## 8. Clinical Resistance to Small Molecule Inhibitor Targeting RTKs: Advances and Pitfalls of Targeted Therapy

Today, anticancer therapies including radiotherapy, chemotherapy, combination, surgery, immunotherapy, and laser therapy are well reported; however, there is still dire need to discover a specific target in solid tumors [108,109,110,111]. Although, several new targets such as carbonic anhydrases, BCl2, Bax, TEAD, and HDACs are being explored to overcome drug resistance/drug failure in cancer management [112,113,114]. Additionally, multiple drug resistance due to ablation, mutation, and genomic instabilities is also considered one of the biggest challenges in the clinical model of several cancers [40,41,42,115]. Drug resistance consists of primary and secondary subtypes. Primary resistance is defined as patients who do not reach a stable disease under six months of an initial clinical response. Elsewhere, it shows primary resistance to imatinib is particularly manifest in patients with a KIT exon nine mutation [116]. PDGFRA also depicts primary mutation at kinase domain and lead conformational changes by which it repones primary resistance to imatinib [117]. Similarly, mutation in KRAS is also associated with primary resistance in treatment with EGFR inhibitors [41,42].

Secondary resistance to RTK inhibitors is particularly common in individuals who have been treated with kinase inhibitors [118]. Numerous cellular programs have been modulated due to resistance phenotype changes in tumor cells [118]. Structural changes in the kinase domain of mutated RTKs is the most common cause of secondary resistance. Hence, the catalytic activities of kinase are suppressed due to being unable to bind and inhibit RTKs. KIT and EGFR are always associated with secondary mutations and more than 45% of patients have acquired resistance to imatinib, erlotinib, and gefitinib [119,120]. These mutational changes on RTKs directly affect the clinical response to small molecule inhibitors as well as drugs. Additionally, both primary and secondary resistance significantly change the IC_50_ value of RTKs inhibitors or drugs. The Genomics of Drug Sensitivity in Cancer (GDSC) is an online database system by which we can obtain big information on drug sensitivity and resistance, especially several cancer cells with their drug responses. Interestingly, we showed that most RTKs inhibitors are efficient in halting cancer progression by targeting a particular kinase with a fabulous IC50 dose (Table 3).

## 9. Conclusions and Perspectives

Over the last quarter century, we have made significant advances in our knowledge of RTK signaling. Basic research associated with these receptors’ genetics, cellular biology, biochemistry, and structural biology has resulted in a detailed understanding of how this protein family operates. These discoveries have resulted in the creation of a slew of potential medicines, serving as prime instances of laboratory-driven translational research. Similarly, clinical studies of RTKs in several disorders have offered significant mechanistic understanding (i.e., reverse-translational research), allowing for further refinement of treatment methods. The chemical processes behind RTK activation have similar motifs but differ significantly in their details. Only about half of the RTK families are completely known. Exploring other families will undoubtedly yield many new insights regarding RTK activation and signaling in the future. The relevance of direct receptor crosstalk or heterodimerization for signaling specificity remains unknown at the receptor molecule level, as does the precise function performed by intracellular trafficking. Another critical problem for the future is gaining quantitative knowledge of these challenges. RTKs inhibitors have been proven as an effective treatment option for several types of cancer. However, resistance of several RTKs inhibitors towards several cancer cells seems to be a critical obstacle in the successful therapy of cancers. Hence, the development of new effective inhibitors is still required to overcome drug resistance hurdles. Although genetic and pharmacological methods have identified many signaling components in RTK networks, putting these components into mechanistic and quantitative settings is the next significant hurdle. This knowledge might pave the way for new approaches to developing effective therapies for cancer and other illnesses caused by active RTKs.

## Figures and Tables

**Table 1:** Major RTKs in human diseases and their effective inhibitors in clinical trial.

RTK Class	Targets	Inhibitors or FDA Approved Drugs	Predicted Localization Based on Proteinatlas.org	Distribution in Tissue or Organs	Diseases	Reference
ErbB/ EGFR	ErbB1, Her2, Her3, Her4	panitumumab, cetuximab, gefitinib, erlotinib, afatinib, and lapatinib	Cell membrane, cell junctions, secreted	Placenta, most of organs	BRCA, GLIOMA, inflammation, cardiac, etc.	[1,2,62]
IGFR	Insulin, IR, IGF-1	Linsitinib, BMS-754807, XL-228, AXL1717, Masoprocol	Secreted plasma, transmembrane, vesicles	Liver, muscle, pituitary, and hypothelamus cells	Cancer and diabetes	[63,64]
PDGFR	PDGFR-*α* PDGF-β	Nilotinib, Sunitinib, Dasatinib, Pazopanib, Vatalinib, Axitinib	Intracellular membrane, Golgi apparatus, vesicles, nucleoplasm, plasma membrane	Brain, placenta, cervix, kidneys etc.	Cancer, Kosaki syndrome, myofbromatosis, aneurysms, premature aging syndrome, brain calcification	[65]
VEGFR	VEGFRs	Bevacizumab, Sorafenib, Sunitinib, Lenvatinib, Pralsetinib	Intracellular, membrane secreted to blood	Lung, Liver, Blood	Cancer, brain *diseases,* ophthalmic *diseases*	[66]
FGFR	FGFRs, aFGF, FGFs	AZD4547, BGJ398, JNJ42756493, PD173074	Intracellular, plasma membrane, ER, etc.	Most of all body organs	Cancer, kidney, lungs, skeletal muscle, heart, liver-related diseases	[8,67]
NGFR	trkA, trkB, and trkC	Ro 08-2750	Cytosol and plasma membrane	Lymph node, testis, liver, kidneys, colon	Cancer, wound healing, neuronal diseases	[68]
HGFR	c-Met, HGF, MET	K252a SU11274 PHA-665752 ARQ197 SGX523	Plasma membrane and cytosol; is predicted to be secreted	Epithelial, endothelial, neurons, hepatocytes, hematopoietic, melanocytes And all body organs	Cancer, autism, cardiac dysfunctions	[69]
EphR	EPHs, Ephrins	MEDI-547 KB004 XL647 JI-101	Intracellular and membrane	Brain, lung, endocrine tissue, and other organs	Cancer, brain, and neuronal diseases	[70]
*AXL*R	AXL UFO	Bemcentinib R428 TP-0903	Plasma membrane, vesicles, and actin filaments	Muscles, lungs, kidney, colon, and other organs	Cancer, encephalomyelitis, cardiac infraction	[48,71]
RETR	RETS	Alectinib Cabozantinib Lenvatinib Ruxolitinib	Golgi apparatus, cytosol, and plasma membrane	Endocrine, lungs, bone, skin, colon, and other organs	Cancer	[72]
LTKR	LTK ALK TYK1	Ceritinib, alectinib, RO5424802, AP26113, ASP3026, TSR-011, PF-06463922, RXDX-101, X-396, and CEP-37440	Intracellular, membrane, vesicles	Enterocytes, mucus-secreting cells, Ito cells, and Kupffer cells	Cancer	[18,19]
ROSR	ROS1	Crizotinib Entrectinib Lorlatinib Ceritinib Cabozantinib	Vesicles, intracellular, membrane	Lung Epididymis Cerebral cortex Olfactory region	Cancer	[19]

**Table 2:** Under top 40 selective RTKs inhibitors (drugs) in clinical trial for multiple cancers.

TKs	Clinical Trial Status	Conditions/Cancer	Interventions	References
EGFR	Completed Phase_II	Non-Small Cell Lung Cancer	Drug: sorafenib (Nexavar)	[20]
VEGF	Completed Phase-I	Refractory or Recurrent Solid Tumors	Drug: Axitinib	[77]
Multiple TKs	Completed Phase 2	Platinum Refractory Epithelial Ovarian Cancer, Primary Cancer of the Peritoneum, Cancer of the Fallopian Tube	Drug: Sunitinib, Drug: SUNITINIB	[78]
VEGF	Completed Phase 1 Phase 2	Pancreatic Cancer	Drug: Vatalanib, Drug: Gemcitabine	[79]
EGFR	Completed Phase 2	Non-small Cell Lung Cancer	Drug: Aprepitant, Desloratadine, Placebo of aprepitan, Placebo of desloratadine	ClinicalTrials.gov Identifier: NCT02646020
VGFR	Completed	Distal Urethral Cancer, Proximal Urethral Cancer, Recurrent Bladder Cancer, Recurrent Transitional Cell Cancer of the Renal Pelvis and Ureter, Recurrent Urethral Cancer, Stage IV Bladder Cancer Transitional Cell Carcinoma of the Bladder Urethral Cancer Associated With Invasive Bladder Cancer	Drug: pazopanib hydrochloride	ClinicalTrials.gov Identifier: NCT00471536
VGFR	Completed Phase 1 Phase 2	Metastatic Renal Cell Cancer	Drug: Everolimus	[80]
MET and VEGFR2	Completed	Neoplasms, Head and Neck	Drug: GSK1363089 (foretinib)	ClinicalTrials.gov Identifier: NCT00725764
EGFR	Completed Phase 3	Non-small Cell Lung Cancer	Drug: Gefitinib, Gemcitabine + Carboplatin	[81]
VEGFR, PDGFR, FGFR and c-Kit	Completed Phase 2	Advanced Malignancy	Drug: Anlotinib	ClinicalTrials.gov Identifier: NCT04216082
VEGFR	Completed Phase 3	Metastatic Renal Cell Carcinoma	Drug: RAD001, Drug: Placebo	ClinicalTrials.gov Identifier: NCT00410124
Multi TKs	Completed Phase 4	Colorectal Neoplasms, Gastrointestinal Stromal Tumors	Drug: Esomeprazole 40 mg concomitantly, Esomeprazole 40 mg before, Regorafenib 160 mg or 120 mg	ClinicalTrials.gov Identifier: NCT02800330

**Table 3:** Drug resistance results based on GDSC drug treatment data and the substructure mutations.

Kinase/Target	Drug (Inhibitor)	Cancer Code	Cancer Name	AvgIC50(G1)	AvgIC50(G2)
ALK	Alectinib	DLBC	B_cell_lymphoma	37.2207	29.0609
ALK	Crizotinib	DLBC	B_cell_lymphoma	18.5283	10.8294
BMX	QL-XII-47	LAML	acute_myeloid_leukaemia	5.7441	1.6628
BRAF	AZ628	LUAD	lung_NSCLC_adenocarcinoma	22.0213	9.4282
BRAF	HG6-64-1	LUAD	lung_NSCLC_adenocarcinoma	17.6081	16.644
DDR1	QL-XI-92	MM	myeloma	17.0624	13.4321
DDR1	QL-XI-92	UNCLASSIFIED	anaplastic_large_cell_lymphoma	23.6282	3.7261
EGFR	Afatinib	LUAD	lung_NSCLC_adenocarcinoma	0.9592	0.0191
EGFR	AST-1306	LUAD	lung_NSCLC_adenocarcinoma	1.1852	0.2463
EGFR	AZD8931	LUAD	lung_NSCLC_adenocarcinoma	2.622	0.0531
EGFR	Cetuximab	LUAD	lung_NSCLC_adenocarcinoma	69.9293	6.8975
EGFR	CI-1033	LUAD	lung_NSCLC_adenocarcinoma	2.1347	0.2929
EGFR	CUDC-101	LUAD	lung_NSCLC_adenocarcinoma	1.3804	0.165
EGFR	Erlotinib	UNCLASSIFIED	Burkitt_lymphoma	20.2694	10.3597
EGFR	Foretinib	UNCLASSIFIED	Burkitt_lymphoma	3.3933	1.2532
EGFR	Gefitinib	LUAD	lung_NSCLC_adenocarcinoma	9.8753	0.0121
EGFR	Gefitinib	UNCLASSIFIED	Burkitt_lymphoma	15.191	9.6238
EGFR	Lapatinib	UNCLASSIFIED	Burkitt_lymphoma	13.9917	9.9664
EGFR	Pelitinib	LUAD	lung_NSCLC_adenocarcinoma	0.0604	0.0543
EGFR	PF-00299804	LUAD	lung_NSCLC_adenocarcinoma	1.6263	0.2417
EGFR	Sapitinib	LUAD	lung_NSCLC_adenocarcinoma	3.5254	0.0979
ERBB4	AST-1306	LAML	acute_myeloid_leukaemia	4.8215	4.4793
ERBB4	AST-1306	UNCLASSIFIED	lung_NSCLC_carcinoid	6.282	5.0641
ERBB4	CI-1033	SKCM	melanoma	23.8639	8.6932
ERBB4	CI-1033	UCEC	endometrium	8.9198	6.6994
ERBB4	PF-00299804	UCEC	endometrium	17.4219	10.298
FLT1	Cediranib	UNCLASSIFIED	anaplastic_large_cell_lymphoma	5.8298	4.1008
FLT1	Linifanib	UNCLASSIFIED	ovary	11.8566	9.8702
FLT1	Midostaurin	BLCA	bladder	1.7165	1.4931
FLT1	Tivozanib	BLCA	bladder	1.8598	1.5754
FLT1	Tivozanib	UNCLASSIFIED	ovary	2.5587	1.3064
FLT3	Lestaurtinib	LUAD	lung_NSCLC_adenocarcinoma	6.5216	5.5234
FLT3	Ponatinib	LUAD	lung_NSCLC_adenocarcinoma	5.3224	4.8343
FLT3	Quizartinib	LUAD	lung_NSCLC_adenocarcinoma	20.1255	14.9548
IGF1R	BMS-754807	UNCLASSIFIED	fibrosarcoma	25.2865	1.4107
IGF1R	GSK1904529A	UNCLASSIFIED	fibrosarcoma	70.7571	27.3638
JAK1	AZD1480	UNCLASSIFIED	endometrium	3.9166	0.4414
JAK1	JAK1_8709	UNCLASSIFIED	endometrium	67.0713	33.19
JAK1	JAK_8517	GBM	glioma	173.1292	56.1354
JAK1	JAK_8517	UNCLASSIFIED	endometrium	176.2048	2.4967
JAK3	WHI-P97	UCEC	endometrium	98.2843	80.5835
KDR	Linifanib	COREAD	large_intestine	18.5488	16.919
KDR	Motesanib	PRAD	prostate	30.6555	12.079
KDR	Ponatinib	UNCLASSIFIED	lung_NSCLC_carcinoid	6.5232	5.613
KIT	Dasatinib	LAML	acute_myeloid_leukaemia	12.8718	7.8024
KIT	Imatinib	LAML	acute_myeloid_leukaemia	23.4979	15.9051
KIT	Sorafenib	LAML	acute_myeloid_leukaemia	40.2854	4.4655
KIT	Sunitinib	LAML	acute_myeloid_leukaemia	35.55	6.7039
LCK	JW-7-24-1	ALL	lymphoblastic_leukemia	1.4246	0.6944
LCK	Staurosporine	ALL	lymphoblastic_leukemia	0.0532	0.0288
LTK	HG-5-113-01	PRAD	prostate	7.8398	4.7173
NTRK1	GW441756	UCEC	endometrium	16.7441	11.9791
NTRK1	GW441756	UNCLASSIFIED	B_cell_leukemia	38.3105	24.7002
NTRK3	AZD1332	ALL	lymphoblastic_T_cell_leukaemia	37.467	20.9519
NTRK3	Lestaurtinib	ALL	lymphoblastic_T_cell_leukaemia	0.4766	0.2518
PDGFRA	Ponatinib	COREAD	large_intestine	13.9216	5.3747
PDGFRB	Dasatinib	PRAD	prostate	52.0138	10.5922
PDGFRB	Motesanib	PRAD	prostate	22.6324	13.6836
PDGFRB	Pazopanib	PRAD	prostate	50.5275	40.7679
PDGFRB	Sorafenib	PRAD	prostate	26.2459	26.1382
RET	Cabozantinib	PRAD	prostate	63.4851	12.5383
ROS1	Crizotinib	GBM	glioma	560.1637	42.8829
SYK	BAY-61-3606	KIRC	kidney	319.3293	15.0456

## Data Availability

Not applicable.
